# Adult Stem Cells Spheroids to Optimize Cell Colonization in Scaffolds for Cartilage and Bone Tissue Engineering

**DOI:** 10.3390/ijms19051285

**Published:** 2018-04-25

**Authors:** Leandra Santos Baptista, Gabriela Soares Kronemberger, Isis Côrtes, Letícia Emiliano Charelli, Renata Akemi Morais Matsui, Thiago Nunes Palhares, Jerome Sohier, Alexandre Malta Rossi, José Mauro Granjeiro

**Affiliations:** 1 Nucleus of Multidisciplinary Research in Biology (Numpex-Bio), Federal University of Rio de Janeiro (UFRJ) Xerém, 25245-390 Duque de Caxias, Rio de Janeiro, Brazil; gabrielaskronemberger@gmail.com (G.S.K.); isistcortes@gmail.com (I.C.); leticiacharelli@gmail.com (L.E.C.); rezinhaakemi@gmail.com (R.A.M.M.); 2 Laboratory of Tissue Bioengineering, National Institute of Metrology, Quality and Technology (Inmetro), 25250-020 Duque de Caxias, Rio de Janeiro, Brazil; jmgranjeiro@gmail.com; 3 Post-graduation Program in Biotechnology, National Institute of Metrology, Quality and Technology (Inmetro), 25250-020 Duque de Caxias, Rio de Janeiro, Brazil; 4 Post-graduation Program of Translational Biomedicine (Biotrans), Unigranrio, Campus I, 25071-202 Duque de Caxias, Rio de Janeiro, Brazil; 5 Brazilian Center for Physics Research, Xavier Sigaud 150, 22290-180 Urca, Rio de Janeiro, Brazil; thiagonup@gmail.com (T.N.P.); alex.mrossi@gmail.com (A.M.R.); 6 Laboratory of tissue biology and therapeutic engineering—UMR 5305, CNRS, 69007 Lyon, France; jerome.sohier@ibcp.fr; 7 Laboratory of Clinical Research in Odontology, Fluminense Federal University (UFF), 24020-140 Niterói, Brazil

**Keywords:** adult stem cells, spheroids, scaffolds, building-blocks, biofabrication

## Abstract

Top-down tissue engineering aims to produce functional tissues using biomaterials as scaffolds, thus providing cues for cell proliferation and differentiation. Conversely, the bottom-up approach aims to precondition cells to form modular tissues units (building-blocks) represented by spheroids. In spheroid culture, adult stem cells are responsible for their extracellular matrix synthesis, re-creating structures at the tissue level. Spheroids from adult stem cells can be considered as organoids, since stem cells recapitulate differentiation pathways and also represent a promising approach for identifying new molecular targets (biomarkers) for diagnosis and therapy. Currently, spheroids can be used for scaffold-free (developmental engineering) or scaffold-based approaches. The scaffold promotes better spatial organization of individual spheroids and provides a defined geometry for their 3D assembly in larger and complex tissues. Furthermore, spheroids exhibit potent angiogenic and vasculogenic capacity and serve as efficient vascularization units in porous scaffolds for bone tissue engineering. An automated combinatorial approach that integrates spheroids into scaffolds is starting to be investigated for macro-scale tissue biofabrication.

## 1. Background

“Top-down tissue engineering” approaches aim to restore the functions of damaged or lost tissues using biomaterials as scaffolds [1]. An ideal biomaterial must mimetize the physical and chemical properties of a tissue extracellular matrix guiding proliferation, migration, and differentiation of stem cells [2]. Smart biomaterials refer to stimulus-responsive materials that can undergo controlled modification of their properties through stimulus such as temperature, pH, moisture, and electric or magnetic fields [3]. The advantage of using smart biomaterials over conventional ones relies on their increased cell affinity and tissue repair. Indeed, temperature and pH-responsive smart biomaterials have been applied as delivery agent for drugs, DNA, antibiotics, and growth factors [4,5]. Nonetheless, the use of thermos-responsive coatings for tissue engineering is beneficial due to their ability to harvest the cell sheet without enzymes (e.g., trypsin), maintaining intact the structure of the extracellular matrix produced by the cells [6]. Smart hydrogels, commonly used as bioink for bioprinting technology, may also respond to temperature-induced properties change [7]. However, in scaffold-based approaches cells in suspension are not distributed homogeneously; besides, large-scale tissue construction is impaired [8]. In order to solve these issues, the scaffold-free approach using adult stem cells is being developed based on organogenesis process recapitulated in vitro [9]. In scaffold-free approaches, cells are arranged directly with each other, re-creating a functional and ordered three-dimensional (3D) structure named as spheroids [10,11].

Recently, spheroids have been used in the following ways (1) in tissue engineering, as a model of organogenesis, better known as developmental engineering [12,13,14]; (2) they have been seeded into biomaterials to improve tissue regeneration in vivo [15]; and (3) they have been used as building blocks for bioprinting and bioassembly approaches [16]. The aim of this review is to summarize the self-assembly process and molecular biology of spheroids, as well as their use in developmental tissue engineering and their association with biomaterials, revealing an innovative perspective of the biofabrication line, in which spheroids can be automated and seeded on the biomaterials’ surface for large scale tissue engineering.

## 2. Scaffolds in Top-Down Tissue Engineering

### 2.1. Cartilage

Cartilage is an avascular and aneural tissue with a low metabolic rate, representing a challenge for regeneration approaches. Therefore, lesions related to cartilage impair life quality of an actual growing age population. Deterioration of this tissue is usually treated with drugs, physical therapies, and, in many cases, surgery. In this context, top-down tissue engineering can be considered as an alternative route for treatment, once a scaffold that can support cell growth and differentiation is developed that allows cartilage repair [17].

There are two main approaches for cartilage engineering: hydrogel and solid scaffold. Several biomaterials have been investigated for the production of injectable hydrogel, which include natural and synthetic biomaterials [18]. Hydrogels have several advantages, including a network that promotes cell adhesion, migration, and proliferation. Such benefits are provided due to 3D network microenvironment that mimics the extracellular matrix and is capable of delivering nutrient and growth factors [19].

The extracellular matrix of cartilage is extremely complex and is composed mainly of collagen type II and proteoglycans [20]. In this context, methodology to produce scaffold using hyaluronic acid and collagen type II coupled with transforming growth factor-β1 into the hydrogel has been extensively developed. In this system, chondrocytes maintain their viability, as well as their chondrocytic properties [21]. Another great advantage of using injectable hydrogel is due to its ability to adjust to the shape of irregular defects. For example, [22] performed decellularization and enzymatic digestion from porcine meniscus to obtain a meniscus-derived hydrogel. Mouse subcutaneous implantation showed excellent biocompatibility, holding promise for future in vivo studies on repair of meniscus.

The use of hydrogels also represents a minimally invasive methodology that can be performed by injection or arthroscopy [23]. In clinical scenario, hyaluronic acid has widely used; however, an important drawback is the limited durability in joint cavity. As an alternative, in vivo studies using osteoarthritis-induced rabbit model suggest that chitosan hydrogel prevented cartilage degradation and synovial membrane inflammation [24].

Similar to hydrogel, solid scaffolds may also be of natural and synthetic derivatives. Collagen scaffold and decellularized cartilage are one of the main natural solid scaffold. Decellularization is mainly important because it contains bioactive signals from native cartilage that guide cellular events such as adhesion and proliferation, and, above all, it has the capacity to be guiding cellular differentiation. In addition, the extracellular matrix contributes to the maintenance of its mechanical properties, which is mainly related to the arrangement of the collagen fibers [25]. Utomo and collaborators (2015) showed the potential of decellularized ear cartilage scaffolds from in vitro studies [26]. Kang and collaborators (2014) showed full thickness repair in rabbit’s femur after adipose stem cells (ASC)-loaded decellularized cartilage extracellular matrix scaffolds [27]. However, donor site morbidity for autologous and allogeneic decellularized cartilage represents an important disadvantage [28].

Recent studies have revealed that collagen scaffold associated with bioactive molecules and cells is capable of generating an efficient engineered tissue. In this context, it has recently been demonstrated that ASCs are capable of acquiring a chondrocytic phenotype when associated with collagen-based scaffold and bioactive factors [29]. In order to mimic the interface bone and cartilage present in the knee joint, collagen sponge-incorporating cartilage and bone matrix-component chondroitin sulfate/hydroxyapatite (HA) composite has been developed [30].

In conclusion, cartilage tissue engineering is a promising approach; however, some problems are still unsolved, such as a stable chondrocyte phenotype from in vitro-differentiated MSCs or ASCs and the microarchitecture of the ideal scaffold.

### 2.2. Bone

Progressively, the population is becoming a victim of bone critical defects because of trauma and illness related to this tissue. The current treatment is autologous or heterologous grafting [31]. On the other hand, this worldwide problem has stimulated the development of biomaterials as an alternative, to be used as implanted scaffolds. As with any other biomaterial, the golden standard properties for bone regeneration are (1) biocompatibility, (2) bioactivity, (3) mechanical resistance, and (4) having a porous structure to allow cells to grow and proliferate into a new, healthy, bone-like tissue [32].

Several synthetic scaffolds can be used for bone tissue engineering because they have the basic properties already mentioned. However, all types of materials have advantages and disadvantages that must be considered before application. For example, inorganic bioceramics are bioactive and biodegradable; at the same time, these cannot be classified as suitable for load-bearing scaffold applications [31].

Another type of scaffold generally used for bone tissue engineering is polymers, which can be natural or synthetic. Natural polymers such as fibrin, hyaluronic acid, chitosan, and collagen have a nice biocompatibility and osteoinduction properties. Otherwise, synthetic polymers such as polyanhydride, polypropylene fumarate (PPF), polycaprolactone (PCL), polyphosphazene, polylactic acid (PLA), polyether ether ketone (PEEK), and poly(glycolic acid) (PGA) have controlled biodegradation. Due the flexibility properties, polymers can be fabricated at low cost using different complex shapes. Besides, polymers can be used as smart materials to deliver soluble molecules [33].

Metal materials have a long history in bone tissue engineering. The principal advantages of metal scaffolds, such as titanium, stainless steels, and cobalt, are their excellent biocompatibility, mechanical strength, and elevated corrosion resistance. However, implanted scaffolds produced from metal materials are generally stiffer compared to live bones, leading to eventual failure of implants [34].

Bioceramics are used in approaches to repair bone with the intention of replacing metal materials. Ceramics, such as beta-tricalcium phosphate (TCP), HA, and dicalcium phosphates, are biocompatible, have excellent resistance to corrosion, and have already been proven to have elevated bioactivity in vivo. However, their principal disadvantages are low fracture toughness, fragility, and stiffness [35].

Composites can include two or more materials from all three groups mentioned: (1) polymers; (2) metals; and (3) ceramics, natural or synthetic [36], with the aim of combining properties. The most current composite material used is the inorganic-organic type, because it combines ductility of polymers with elevated stiffness of inorganic components, creating a material with better mechanical properties and a better biodegradation rate [37]. For example, Yang and colleagues (REF) achieved full repair using collagen type I and porous TCP scaffolds seeded with ASCs in a rabbit model [38].

Classical or top-down tissue engineering combines cells with scaffolds. Mesenchymal stem cells (MSCs) or ASCs may be mixed with injectable scaffolds as hydrogels or seeded on a porous composite biomaterial. Ex vivo amplification is then followed by direct transplantation or by pre-differentiation before transplantation. A recent strategy proposed for bone tissue engineering is a pre-differentiation to cartilage as a template for endochondral bone repair after transplantation [39].

Most studies involve the use of ceramic and composite materials alone or with MSCs or ASCs. Calabrese and collaborators (2017) developed a composite scaffold made of collagen-(HA) and characterized its regenerative properties in vivo after subcutaneous implantation in mice [40]. The efficiency of angiogenesis and osteogenesis was evaluated by Fluorescent Molecular Tomography (FMT) in vivo. The main result found was that scaffolds seeded with ASCs had improved mineralization and vascularization.

In conclusion, the use of scaffolds, mainly composites and ceramics, as an approach for bone tissue engineering is very promising for substitute bone grafts. However, there are some challenges, such as, for instance, the (1) difficulty of making the process (cells and scaffolds) industrially scalable to attend population demand, (2) cost of a perfect design and production of constructs, and (3) diffusion of nutrients inside the scaffolds [40].

## 3. Developmental Tissue Engineering

### 3.1. Spheroids

Since a discovery of the cell as a basic tissue unit, two-dimensional (2D) cell culture has been routinely used by several laboratories. However, 2D culture does not replicate the tissue morphology for more robust and profound studies on cellular biology and physiology, because it does not mimic the native tissue microenvironment.

Spheroids are formed through a process named self-assembly. Initially, cells stay in direct contact with each other to form aggregates due to long extracellular matrix fibres with multiple Arginylglycylaspartic acid (RGD) motifs interacting tightly with integrin on cells membranes surfaces. The direct cell to cell contact due to these initial aggregation results in upregulation of N-cadherin expression. The accumulation of N-cadherin in membrane surface drives compaction, resulting in spheroids [41].

Spheroids optimize intracellular signaling, improving differentiation process, which in turn enables cells to be organized into a more similar structure of tissues in vivo. Moreover, in spheroids, receptors and adhesion molecules are more naturally spread. In 2D culture, cells are polarized, and their receptors are concentrated in the ventral surface, in which cells interact directly with flask culture plastic [42].

### 3.2. Organogenesis and Spheroids

Spheroids were initially used as embryonic or tumor models. Moscona and Moscona were pioneers using the self-assembly of cell suspensions from organ rudiments of the early chick embryo. The authors discovered that it was possible to produce tissue-like aggregates showing the ability to recapitulate characteristics of tissue in vivo [43].

Currently, spheroids have been used in tissue engineering for drug screening and regenerative medicine approaches. Multilineage differentiation potential from MSCs is significantly enhanced in spheroids culture, mainly due to intensive cell-cell and cell-extracellular matrix contacts established in 3D microenvironment of spheroids [44]

MSCs self-assembly into spheroids improves expression of anti-inflammatory protein tumor necrosis factor (TNF)–alpha stimulated gene/protein 6 (TSG-6), and the paracrine secretion of angiogenic factors, including vascular endothelial growth factor (VEGF), basic fibroblast growth factor (bFGF), and angiogenin [45,46], representing attractive vascularization units. In this context, MSCs spheroids were applied in several preclinical studies, involving different animal models. The main in vivo studies were done with the intention of repairing osteochondral diseases, cardiovascular disorders, and wound healing [47].

More importantly, spheroids provide a controlled spatial organization of MSCs and ASCs, recapitulating important events of morphogenesis—a field known as developmental engineering [48]. In fact, the nomenclature of spheroids and organoids can be misunderstood. Organoid has been defined as a 3D structure derived from stem cells (embryonic, neonatal, or adult source) resembling their in vivo tissue counterpart and mimicking at least one function of tissue or organ [49,50]. For example, Kale and collaborators (2000) showed crystalline human bone from spheroids [51], which was later categorized as organoids [49]. Therefore, we can postulate that MSC spheroids showing at least one function of tissue or organ (e.g., crystallization in bone) could be categorized as organoid.

Spheroids from ASCs were used to promote bone [52] and cartilage [53] regeneration successfully. For example, our research group has recapitulated in vitro chondrogenic events using spheroids from ASCs (manuscript in preparation) and cartilage progenitor cells. Cartilage progenitor cells spheroids represent an interesting model, since they present gene upregulation of Trio SRY-Box (SOX) up to 200 times compared to monolayer [54].

Spheroids and organoids have revolutionized the understanding of cellular behavior [55,56,57] providing a relevant tissue microenvironment closer to human physiology. Many “omics” research studies, primarily focused on the analysis of secreted proteins, allow the identification of protein biomarkers for cellular modifications such as cell differentiation or disease progression. The secretome (secreted proteins) are an important class of proteins that control and regulate a range of physiological and biological processes, becoming a source of relevant biomarkers, including therapeutic target findings [58].

The search for biomarkers in secretome (proteomic of culture medium) using spheroids is a promising approach for identifying new molecular targets for diagnosis and therapy. For example, our research group is currently investigating the secretory capacity using proteomic of ASC spheroids induced into chondrocytes to identify possible biomarkers for a stable chondrocyte phenotype (manuscript in preparation). Recently, Santos and collaborators detected higher amounts of several growth factors in culture medium of umbilical cord tissue-derived MSCs compared to monolayer [59].

## 4. Scaffolds and Spheroids

The association of spheroids with scaffolds is a fascinating approach for tissue engineering. Scaffolds must recreate extracellular matrix in which cells in spheroids can adhere, proliferate, and differentiate [15]. Moreover, the scaffolds should be designed to retain the spheroids in vivo to promote repair, vascularization, and, finally, regeneration of injured tissue. The spheroids have the intrinsic capacity to fuse to each other, mimicking the natural tendency of embryonic tissues during morphogenesis [60].

This innovative approach that integrates spheroids into scaffolds has been investigated in several studies. Huang and colleagues demonstrated the association between ASC spheroids into poly(lactide-co-glycolide (PLGA) scaffold for chondral defect using rabbit as animal model. The quality of in vivo neo cartilage was proportional to substrate composition and culture conditions [61]. Ho and collaborators investigated the efficacy of multicellular spheroids compared to cell monolayer seeded into 3D polymeric biodegradable scaffolds. The results suggested that spheroids represent a better tool for recreating carcinogenic microenvironment [62].

Laschke and collaborators (2014) produced undifferentiated and bone-differentiated spheroids of murine ASCs for polyurethane scaffolds seeding [63]. The engineered constructs were implanted into dorsal skinfold chambers in a mice model. Interestingly, scaffolds seeded with one differentiated spheroid exhibited a markedly impaired vascularization in vivo. Ho and collaborators (2017) compared in vitro and in vivo the functions of MSC spheroids and monolayer induced to bone [64]. After 8 weeks of implantation in mice, both materials contained mineralized tissue; however, elevated osteocalcin staining was found in induced spheroids compared to monolayer.

To achieve success, the association between scaffolds and spheroids must rely on four main parameters: (1) The scaffold design should consider the spheroid size, as well as the anatomic implantation site; (2) The final construct should be scalable; (3) Spheroids must be able to interact with each other. The scaffold architecture should not interpose between them; and (4) The process of scaffold fabrication should not impair its functionalization. For example, it must be permitted to carry molecules for cell survival or differentiation.

### 4.1. Spheroids Seeded into Nanofibers

Polymeric fibers have been a major target for cell delivery strategies, mainly due to their wide cell adhesion surface and low volume, as well as high porosity [65]. These characteristics allow cell growth, migration, and differentiation, and enable a greater interaction of cells with polymeric fibers [66]. Nanofiber scaffolds were developed in order to create a system similar to the natural extracellular matrix. Cells that dwell in tissue are inserted inside a 3D matrix composed of ambiguous collagen content [67].

**Figure 1:**
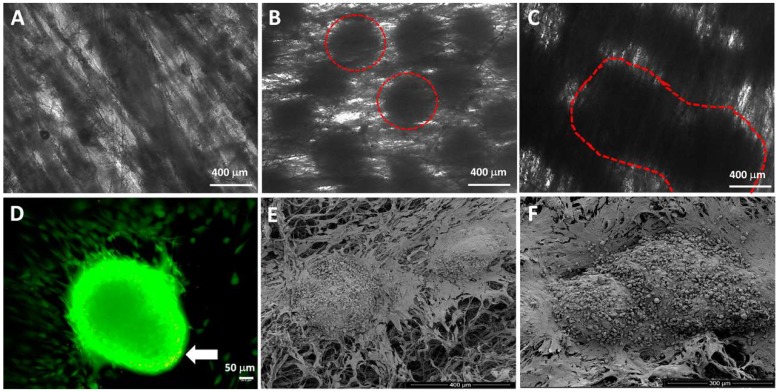
Association of ASC spheroids induced for chondrogenic pathway in PCL nanofibers. (**A**) Aligned PCL nanofibers, (**B**) induced ASC spheroids associated with PCL nanofibers, (**C**) initial stage of induced ASC spheroids fusion, (**D**) induced ASC spheroids associated with PCL nanofibers incubated with cytoplasmic calcein (green staining) and ethidium homodimer (red staining) revealed mostly viable cells and few necrotic cells (arrow). (**E**,**F**) scanning electron microscopy of induced ASC spheroids associated with PCL nanofibers showing initial stages of spheroids proximity (**E**) and fusion (**F**).

Our research group has developed an innovative methodology for seeding spheroids in polymer nanofibers. Our aim is a cartilage construct, since ASC spheroids are pre-differentiated in chondrocytes. ASC spheroids differentiated in chondrocytes are seeded in aligned nanofibrillar structures produced by jet-spraying using PCL [68]. This nanofiber was chosen mainly due to its biocompatibility, low degradation rate, and mechanical property. ASC spheroids differentiated in chondrocytes after seeding in aligned nanofibrillar structure showed rapid and homogeneous cell dispersion by material surface (Figure 1B,D). Furthermore, our ASC spheroids fused to each other (Figure 1C), increasing the cell contact of the nanofibers surface with high cellular viability (Figure 1D). On the other hand, ASC spheroids differentiated in chondrocytes after seeding in non-aligned nanofibrillar structure showed a non-homogeneous cell dispersion preventing an efficient cell colonization along nanofibers surface [69]. An efficient spheroid seeding in nanofibers is critical for the retention of cartilage construct in implantation site.

Chua et al. showed that the association of surface modified nanofibers with hepatocyte spheroids results in a functional tissue liver construct capable of secreting albumin, regulating ammonia metabolism and enzymatic activity of the cytochrome P450 [70]. The nanofibers high porosity mimics 3D arrangement of collagen fibers favoring a rapid and homogenous cell distribution along nanofibers surface. In this study, besides the cell distribution, we observed spheroids fusion (Figure 1). On the other hand, Chua et al observed an efficient retention of individualized spheroids due to surface modification of nanofibers. We can assume that the impairment of spheroids fusion favoured the integration of spheroid-nanofiber construct, reaching the engulfment of spheroids by nanofibers. The surface modification of nanofibers should be evaluated according to the desired tissue.

### 4.2. Cell Suspension and Spheroids Seeded into 3D Printed Scaffold

ASC spheroids have already been associated with biomaterials to promote bone regeneration in vivo. The main advantage of this approach is the increase of vascularization due to angiogenic capacity of ASC spheroids [63].

**Figure 2:**
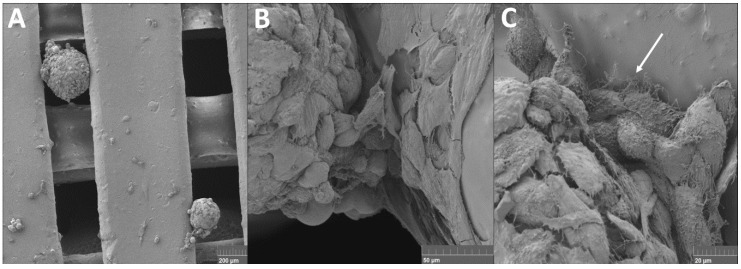
Scanning electron microscope of ASC spheroids associated with 3D PLA/CHA printed scaffold. (**A**) Two spheroids in different areas of scaffold. Bar size: 200 μm; (**B**) Cells of spheroid interacting with the biomaterial surface. Bar size: 50 μm; (**C**) Note the presence of filopodia (arrow) produced by spheroid cells promoting a better interaction with the scaffold. Bar size: 20 μm.

Our research group developed an innovative methodology using a 3D printed composite scaffold made of PLA and carbonate HA (CHA) seeded with ASC spheroids for repair of critical-sized bone defects in vivo. The HA is a ceramic material commonly used in bone tissue engineering once it is considered “osteoinductive” to cells [71,72,73]. Our results showed spheroids interacting with 3D printed scaffold in two different regions (Figure 2A). Cells of spheroids spreaded onto the biomaterial surface (Figure 2B) and produced filopodia structure to improve the association with the biomaterial (Figure 2C).

To our knowledge, this is the first time in scientific literature that 3D printed scaffold has been tested for spheroids seeding. Several studies reported cell suspension seeding in 3D printed scaffolds, in particular for bone tissue engineering [74,75,76,77,78,79,80]. Weinand and collaborators used a TCP scaffold printed with different types of hydrogels mixed with human bone marrow MSCs [76]. The scaffold printed with collagen I hydrogel showed a better osteogenesis in vitro; however, although hydrogels are not mechanically stable, the authors observed that MSCs do not penetrate the scaffold completely. In this study, spheroids interacted with the PLA/CHA printed scaffold, represented by cell spreading in material surface, which may be due to the absence of hydrogel.

Zhang and collaborators (2016) also showed that 3D printed PLA/HA scaffold seeded with human bone marrow promotes cells proliferation and osteogenic differentiation in vitro [77]. A limitation of this study was that cells did not accommodate properly, probably due pore size and the smooth surface of scaffold. Hernandez and collaborators (2017) designed a hybrid system made of printed polycaprolactone mixed with hydrogel composed of alginate, gelatin, and nano-HA and filled with MSCs for large bone defects in vivo [78]. The authors discussed that the presence of nano-HA enhanced the bioactivity and osteoregenerative properties of the scaffold. In this study, the 3D printed scaffold was printed with CHA at nano-scale. We can assume that the presence of CHA attracted cells in spheroids to interact and spread into scaffold surface.

In this study, our results suggested that the impairment of spheroids fusion implies a deeper interaction, resulting in an integrated spheroid-3D printed scaffold construct. Cells migrating from spheroids showed a behavior of dispersion in nanofibers (Figure 1D) in contrast to the spreading observed in 3D printed scaffold (Figure 2B).

## 5. Spheroids, Scaffolds, and Building-Blocks

Spheroids are formed by 3D culture systems and are advantageous compared to other systems. Spheroids have defined geometry and concentric organization of distinct cell populations, which are more related to in vivo [81]. Indeed, due the intrinsic capacity of these spheroids units to fuse to each other (Figure 1C), forming tissues in macro-scale, they are considered as building blocks [8].

The seeding of spheroids into scaffolds, in contrast to cell suspension, allows for the delivery of cells with greater biological features (e.g., enhanced MSCs stemness). Furthermore, the synergism of spheroids and scaffolds (Figure 1 and Figure 2) could promote a successful graft of spheroids in the injured site.

A combinatorial approach that integrates spheroids into scaffolds has been investigated in several studies. Huang and colleagues demonstrated a successful association between ASC spheroids into PLGA scaffold for chondral defect in rabbits [62]. Ho and collaborators investigated the efficacy of spheroids compared to cell monolayer, which were both seeded into 3D polymeric biodegradable scaffolds for cancer therapeutics. Results suggested that spheroids culture might be a better tool for screening cancer therapeutics, mimicking better the carcinogenic microenvironment [62].

Our research group postulated the fabrication of a tissue construct from spheroids within the interlockable solid synthetic microscaffolds denominated as lockyballs [82]. Lockyballs are spheroidal microscaffolds with a diameter of 200 μm architected with hooks and loops to increase the retention capacity of the spheroids at implantation site. ASC spheroids were formed inside lockyballs showing high aggregation capacity in vitro, maintaining master gene expression for chondrogenesis and osteogenesis. This methodology represents an innovative strategy for tissue engineering, since the resulting tissue construct shows a larger area of cells (spheroids) than material and could represent a building-block [83].

### Building-Blocks in Biofabrication

Biofabrication can be defined as “the automated generation of biologically functional products with structural organization from living cells, bioactive molecules, biomaterials, cell aggregates such as micro-tissues, or hybrid cell-material constructs, through bioprinting or bioassembly and subsequent tissue maturation processes” [84]. Biofabrication methods have already been applied with cells and scaffolds to bone tissue engineering and connective and muscle tissue [85,86].

Due to the intrinsic capacity of spheroids to fuse to each other, seeding these building blocks into 3D printed scaffolds might improve the regeneration of critical defects sizes, once it guides the spheroids fusion to the geometry of interest. However, the automated seeding of spheroids onto 3D printed scaffolds to produce a complex 3D construct has not been largely explored. The importance of automated seeding relies on the non-homogeneous distribution of spheroids through the scaffold area, in which they are seeded manually (Figure 1 and Figure 2).

Currently, biofabrication technologies can control the cell distribution with accuracy, creating hybrid cell-biomaterial constructs. Mekhileri and co-works have reported the successfully engineered automated platform that integrates pre-differentiated chondrocyte spheroids bioassembly into a predetermined polymeric scaffold [87]. The system is capable of individualizing the spheroids and forwarding them to the injection module. Once inside the injection module, the spheroids are seeded onto a scaffold that was previously defined based on a 3D positioning system. The data showed long-term cell viability, spheroids fusion, and also typical proteins of extracellular matrix from cartilage.

**Figure 3:**
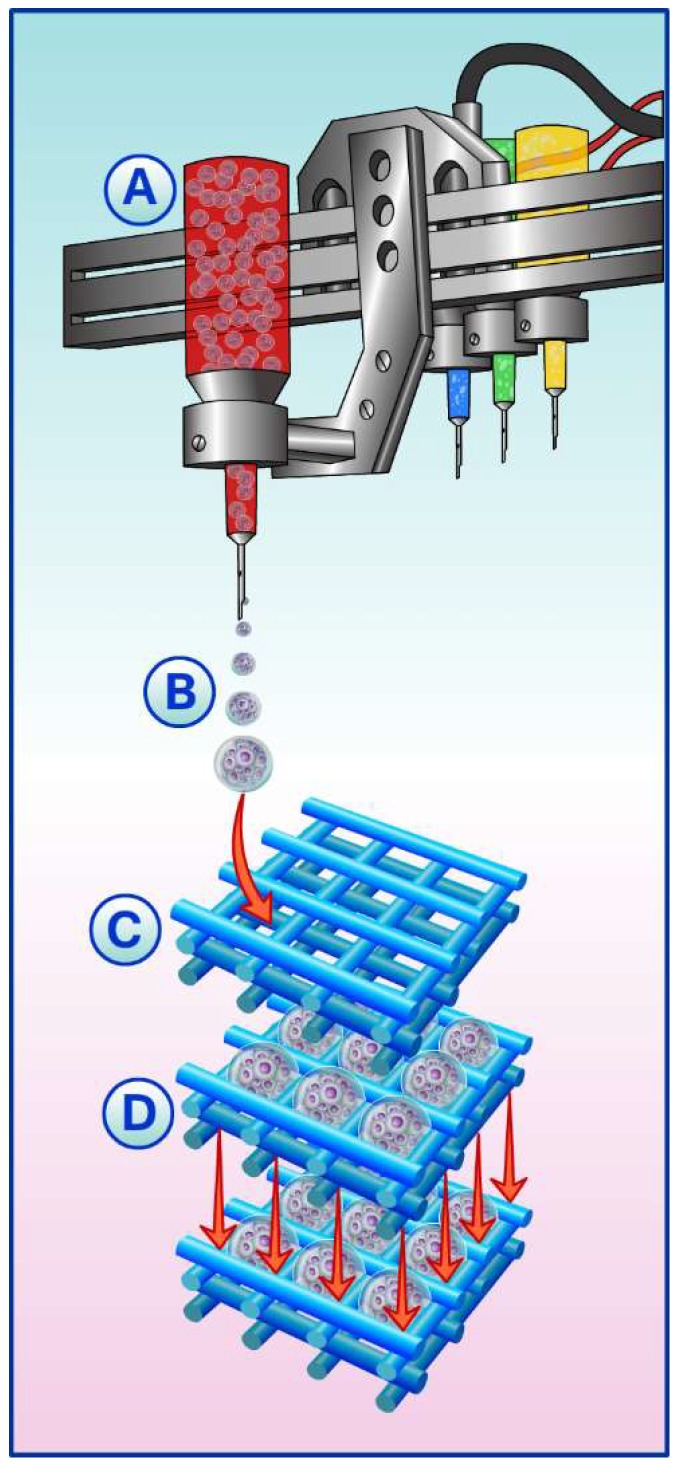
Automated platform for spheroids seeding into 3D printed scaffolds. (**A**) The bioprinter; (**B**) spheroids are dispensed by the bioprinter into the 3D printed scaffolds; (**C**) the 3D printed scaffold; (**D**) the bioprinter dispensing one layer of spheroids for each layer of the 3D printed scaffold. The state-of-art is one spheroid seeded in each spacing of the 3D printed scaffold.

Here, we hypothesized the state-of-the-art for the development of automated platforms capable of precision for spheroids seeding into 3D printed scaffolds based on biofabrication concept (Figure 3).

Another automatization alternative for cells-scaffold seeding is the use of 3D bioprinting. This technology consists of computerized transfer of complex 3D patterns to either assembly biomaterials or cells. In this approach, scaffolds (e.g., hydrogels) act only as structural support, whereas the building block units need fusion to form tissue-like structures [12]. Bhise and co-workers developed a system that integrates a bioprinter and a bioreactor. The main advantage of this technology is monitoring several parameters of spheroids culture in real time (e.g., temperature, CO_2_, infusion of testing drugs). Also, the association with the bioreactor allowed for long-term evaluation of the resulting construct [88].

The development of technologies that promote automated seeding of building-blocks (e.g., spheroids) into scaffolds has become an important key in tissue engineering, since it allows (I) improvement of seeding efficiency, (II) the scale-up of production in a cost-effective manner, (III) standardization of the process, and (IV) the homogeneous distribution of cells or spheroids through the scaffold (mainly drawback of top-down approaches). Hence, the success of biofabrication technologies provides the production of living constructs with more biomimicry with in vivo system, which can be used as valuable tool for disease modeling, drug development, and personalized regenerative medicine.

## 6. Conclusions

Spheroids from adult stem cells can be considered as organoids since, they recapitulate tissue morphogenesis resembling at least one tissue/organ function and are beginning to be used in tissue engineering. Even with recent advances in smart materials, cell suspension seeding into scaffolds still represents a drawback in tissue engineering. Spheroids are a new, developing approach for cell seeding, and, based on our preliminary results in vitro, we can assume that cells migrating from spheroids can disseminate and spread onto the scaffold surface, resulting in an integrated spheroid-scaffold construct.

The comparison between ASC spheroids seeding onto nanofibers and the 3D printed scaffold suggested that the impairment of spheroids fusion implies a deeper interaction. Furthermore, 3D printed scaffold provides a defined geometry according to the size and shape of spheroids, enabling automatized seeding using bioprinting approaches. Pre-induced spheroids from adult stem cells bioprinted onto the 3D printed scaffold is a fascinating approach for future clinical trials, since they can form larger, complex, and functional autologous tissues and organs.
